# The implications of N6-methyladenosine (m6A) modification in esophageal carcinoma

**DOI:** 10.1007/s11033-023-08575-2

**Published:** 2023-08-20

**Authors:** Cheng He, Xiao Teng, Luming Wang, Miaoqi Ni, Linhai Zhu, Jiacong Liu, Wang Lv, Jian Hu

**Affiliations:** 1 Department of Thoracic Surgery, The First Affiliated Hospital, Zhejiang University School of Medicine, Hangzhou, China; 2 Echocardiography and Vascular Ultrasound Center, The First Affiliated Hospital, Zhejiang University School of Medicine, Hangzhou, China; 3 Key Laboratory of Clinical Evaluation Technology for Medical Device of Zhejiang Province, Hangzhou, China

**Keywords:** N6-methyladenosine (m6A), Writers, Erasers, Readers, Diagnosis, Therapy, Esophageal carcinoma

## Abstract

Esophageal carcinoma (EC) is always diagnosed at advanced stage and its the mortality rate remains high. The patients usually miss the best opportunity for treatment because of non-specific symptoms and the survival rates are low. N6-methyladenosine (m6A) the predominant modification in eukaryotic messenger RNA(mRNA), serves vital roles in numerous bioprocess. This chemical modification is dynamic, reversible and consists of three regulators: m6A methyltransferases (writers), demethylases (erasers) and m6A-binding proteins (readers). Recently, a growing number of evidences have indicated relationships between m6A and EC. Whereas, lacking of cognition about the molecular mechanism of m6A modification in esophageal carcinoma. We will focus on the biological function roles of m6A modification in the tumorigenesis and development of EC. Recent studies showed that immunotherapy had a positive impact on EC. The relationship between m6A and immunotherapy in EC deserves further research and discussion. We will also discuss the potential clinical applications regarding diagnosis, treatment and prognosis of m6A modification for EC and provide perspectives for further studies.

## Introduction

Esophageal carcinoma (EC) is one of the most frequent and lethal malignant tumors worldwide. According to the Global Cancer Statistics 2020, approximately 604,000 new cases and 544,000 deaths with the EC patient have been reported each year [1]. The major histological types of EC include esophageal squamous cell carcinoma (ESCC) and esophageal adenocarcinoma (EAC). The consumption of both tobacco and alcohol greatly could increase the risk of the ESCC in a synergism. Some suspected pathogenic factors for the ESCC include pickled vegetables and hot food [2]. The major proportion of esophageal cancer in high-income countries is EAC and the risk factors of the EAC are gastroesophageal reflux disease, Barrett’s esophagus, obesity and smoking etc. [3]. Early diagnosis of the EC becomes possible by using esophagus endoscopy. However, non-specific symptoms of the EC result in the negligence of the EC during early stage because esophagus endoscopy is not a regular item for annual physical check-ups. Hence the best diagnostic opportunity of the EC is often delayed. The onset of dysphagia is associated with the advanced stage of the EC and its 5-years survival is lower than 15%. Although immune checkpoint inhibitors has made great progress in curing the EC, the response to programmed cell death protein 1 inhibitors are different between the ESCC and the EAC. Compared with the EAC, the ESCC has a distinct molecular makeup. There is an urgently demand to develop specific biomarkers for diagnosis and candidate therapeutic target drug to treat the disease. RNA epitranscriptomics is a new frontier of studying RNA modifications and N6-methyladenosine (m6A) is essential in various bioprocess in eukaryotic messenger RNAs (mRNAs). Accumulating researches show that m6A methylation has enormous impact on kinds of diseases. Unfortunately, the underlying mechanisms of m6A methylation in a variety of diseases have not been fully elucidated. In this review, the mechanisms of m6A modifications in the EC are sufficiently discussed, especially on proliferation, apoptosis, cell cycle and therapeutic strategies. Moreover, we will investigate the realistic clinical practice of m6A and the possibilities of capitalizing on m6A for EC early diagnosis and treatment.

## RNA methylation

RNA methylation plays a necessary role in kinds of biological processes, such as gene expression, cellular differentiation and so on. With the development of detection methods in RNA modification, various types of RNA methylation were found, including N6-methyladenosine (m6A), N1-methyladenosine (m1A), N7-methylguanosine (m7G) and 5-methylcytosine (m5C)[4]. RNA methylation occurs in different RNA types. m5C has been found in mRNA, rRNA and tRNA. m7G modification includs transfer RNA(tRNA), ribosomal RNA(rRNA), messenger RNA(mRNA), and MicroRNA(miRNA)[5]. Increasing evidence supports the notion that dysregulation of RNA methylation contributes to a diverse range of human diseases, including cancer and immune system disorders.

### Regulators of m6A

**Fig. 1:**
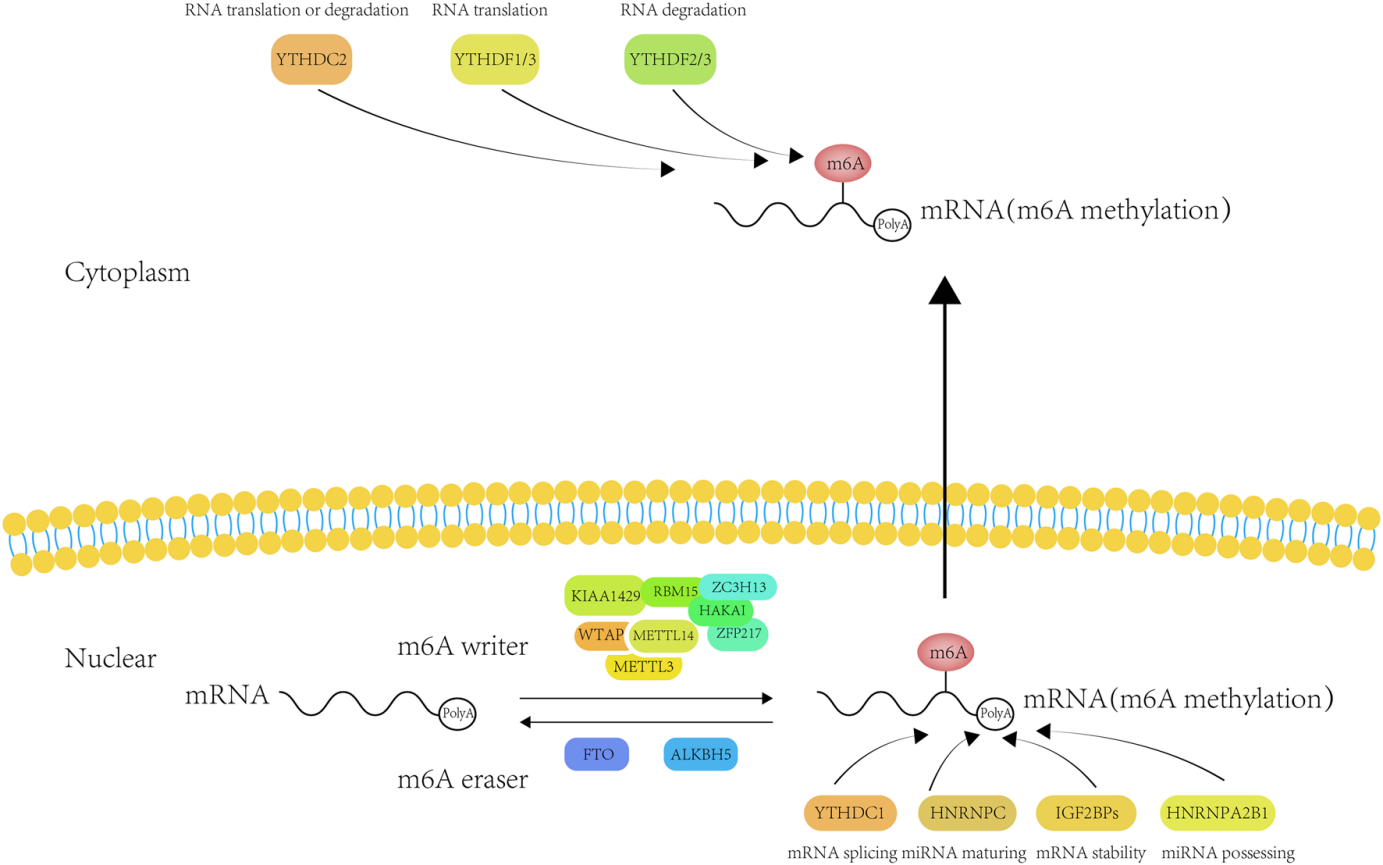
The general mechanism of m6A RNA methylation

m6A modification influences various biological functions as either inhibitor or facilitator in tumor cells reversibly and dynamically. m6A can be catalyzed by RNA methyltransferase (writer), cleared off by the demethylase (eraser) and then interacts with m6A-binding protein (reader). In this part, we are going to elaborate the role of m6A in the occurrence and progression of tumors. Figure 1 visually presents the molecular mechanism of the m6A modification.

### m6A writers

The m6A writers are composed of methyltransferase like protein 3 (METTL3), methyltransferase like protein 14 (METTL14), Wilm’s tumor-associated protein 1 (WTAP), zinc finger CCCH domain protein 13 (ZC3H13), RNA binding motif protein 15 (RBM15), the human homologous gene of Drosophila VIR protein (KIAA1429 or VIRMA) and as well as zinc finger protein 217 (ZFP217) and HAKAI [6–10]. In the combination of METTL3 and METTL14, METTL3 acts as the catalytically subunit while METTL14 plays a role of stabilizer of the complex. The complex has a binding position for S-adenosylhomocysteine (SAH) or S-adenosylmethionine (SAM) [11]. The core ingredient of the m6A methyltransferase is METTL3. WTAP does not possess any catalytic domains and has no catalytic activity on METTL3-METTL14 complex. WTAP serves as a regulator of the METTL3-METTL14 complex and markedly influence the m6A modification depositions. The targeted adenosine residues subunit was methylated following the binding of METTL3-METTL14-WTAP complex and mRNA [12, 13].

Recent year, other proteins have been identified as m6A writers. Yue et al. reported VIRMA mediated selective methylations of mRNA in 3’UTR and near the stop codon by attracting the components METTL3/METTL14/WTAP complex [14]. KIAA1429 deletion could generate aberrant RNA metabolism in folliculogenesis and the maintenance of oocyte competence [15]. KIAA1429 mediates m6A of GATA-binding protein 3 (GATA3) that further regulates cell proliferation and metastasis in liver cancer. In 2016, Patil et al., for the first time, found that RBM15 and RBM15B lead to m6A formation at specific sites of the lncRNA X-inactive specific transcript (XIST) by recruiting the m6A-methylation(m6Am) complex [16]. We also known that ZC3H13 is an m6A writer interacting with an m6A conserved combination including WTAP, Virilizer and HAKAI. And ZC3H13 translocates this compound to the cell nucleus to accelerate m6A methylation [17]. HAKAI is a necessary element required for the diverse subunits of the methyltransferase complex and recruit the core components to promote m6A deposition [18]. Zinc finger protein 217 (ZFP217), up-regulated in kinds of human tumors, is a significant oncogenicity effector [19–22]. Francesca et al. demonstrated that ZFP217 reacted with METTL3 to modulate the m6A deposition [9]. A previous publication revealed that ZFP217 activated m6A demethylase fat mass and obesity-associated protein (FTO) to coordinate m6A mRNA methylation in an m6A-YTHDF2-dependent manner[23].

Recently, newly discovered m6A RNA methyltransferases contains methyltransferase like proteins 5 and 16 (METTL5, METTL16), both can mediate m6A on specific sites of RNAs [24, 25]. Previous studies have found that METTL5 includes a SAM-binding GxGxG motif and a m6A-catalyzing NPPF motif. Bowen et al. found that METTL5 could catalyze m6A modification on the 18S rRNA around region A1832 by means of high-performance liquid chromatography-mass spectrometry (HPLC–MS) analyses [26]. Jessica et al. identified the METTL16 by mass spectrometry, suggesting a hypothetical RNA methyltransferase. METTL16 can modify m6A on U6 snRNA, kinds of ncRNAs, numerous long non-coding RNAs (lncRNAs) and pre-mRNAs [27, 28]. Lately, zinc finger CCHC domain-containing protein 4 (ZCCHC4) was discovered as a novel methyltransferase that could mainly methylate human 28S rRNA and a subset of mRNAs [29].

### m6A erasers

The m6A erasers consist of Fat Mass and Obesity-associated protein (FTO) and AlkB homolog 5 (ALKBH5) which mainly remove m6A transcriptional modification within the nucleus. Thomas et al. first found FTO mediated Fe(II)- and 2OG-dependent nucleic acid demethylation [30]. The crystal structure of FTO protein has two important domains including AlkB-like domain and carboxy-terminal domain. These two domains that interacting with each other corporately catalyze FTO demethylation of 3-methylthymidine (3-meT) in single-stranded DNA and 3-methyluracil (3-meU) in single-stranded RNA [31, 32]. Jia et al. found altering expression level of FTO would change the amounts of m6A [33]. However, the position of FTO as a mainly eraser of m6A has been questioned. Jan et al. found that m6Am is dynamically and reversibly demethylated by FTO and FTO prefer to demethylate substrate of m6Am rather than m6A [34]. Nonetheless, latest study demonstrated that the distribution of FTO in the nucleus and cytoplasm is distinguishing and further affect the way of FTO influencing on kinds of RNA substrates. FTO can bind various genre of RNAs and then mediate m6A or m6Am demethylation on mRNA or small nuclear RNA (snRNA) as well as m1A in tRNA [35]. Relier et al. reported that FTO influenced stem-like properties of colorectal cancer cell through regulating cytoplasmic m6Am demethylation [36]. It seems that FTO is inclined to targets nuclear m6A and cytoplasmic m6A and m6Am in mRNA. Numerous studies demonstrated that FTO could embellish multiple kinds of RNAs, such as lncRNAs, circular RNAs (circRNAs) and mRNAs [37–39]. FTO firstly recognized to be a regulator in obesity and diabetes, performances different functions in cancers. As an m6A demethylase, FTO could regulate multiple functions in cancers, such as cell cycle, apoptosis, proliferation, migration, invasion, stem cell self-renewal and so on. Further studies are needed to explored the detailed functions and concrete mechanisms of FTO. FTO and ALKBH5 demethylases both pertain to the AlkB subfamily of the Fe(II)/2-oxoglutarate (2OG) dioxygenase superfamily. However, ALKBH5 expression selectively demethylates m6A, but not m6Am, indicating that ALKBH5 appears preference for m6A not for m6Am [34]. ALKBH5 reveals a stricter substrate preference and only catalyzes demethylation of m6A containing ssRNAs and the sequence of (Pu[G > A] m6AC [A/C/U]) rather than random sequences [40]. ALKBH5 level is high-expression in various cancer tissues, such as lung cancer and epithelial ovarian cancer [41, 42] but down-regulated in hepatocellular carcinoma, colon cancer and bladder cancer cells [43–45]. In immunotherapy, ALKBH5 has been used to regulate lactate concentration and immune cell accumulation in tumor micro-environment and change the anti-PD-1 therapeutic effect [46]. ALKBH5 not only plays a great role in cancers but also is critical for non-cancer diseases. Li et al. reported that ALKBH5 was a negative regulator in osteogenic differentiation of mesenchymal stem cells [47]. Recently, Zhou et al. proved that ALKBH5 had impact on the pathogenicity of CD4 + T cells during autoimmunity [48] and Zhao et al. demonstrated that ALKBH5 had a adverse effect on post-ischemic angiogenesis [49]. Further investigations should be performed to elucidate the relationship between ALKBH5 and human diseases.

### m6A readers

Currently the well studied “readers” are YTH domain-containing proteins, including YTHDF1-3 and YTHDC1-2. In addition to the YTH reader proteins, there are some other binding proteins also stabilize m6A methylation and generate functional signal, including eukaryotic translation initiation factor 3 (eIF3), staphylococcal nuclease domain-containing 1 (SND1), heterogeneous nuclear ribonucleoprotein C (HNRNPC), heterogeneous nuclear ribonucleoprotein G(HNRNPG), heterogeneous ribonucleoprotein A2/B1(HNRNPA2B1) and insulin-like growth factor 2 mRNA binding protein 1-3 (IGF2BP1-3). Of YTH domain-containing proteins in cancer, YTHDF2 is the firstly identified reader protein and the most extensively studied. YTHDF2 consists of a P/Q/N-rich N terminus and a RNA-binding domain C terminus. C-terminal YTD domain leads to YTHDF2 binding to m6A of target mRNA and N-terminal domain leads to YTHDF2 accelerating the degradation of the targeted mRNA [50]. Notably, the latest study indicated that YTHDF2 was upregulated in prostate cancer and mediated tumor suppressors LHPP and NKX3-1 mRNA degradation in m6A-dependent way [51]. Hou et al. found YTHDF2 expression level is associated with clinical outcome and YTHDF2 deletion promotes hepatocellular carcinoma (HCC) growth, tumor angiogenesis and distant metastasis [52]. YTHDF2 can stabilize MYC mRNA of cancer stem cells and it might be a potential target to regulate MYC signaling pathway in glioblastoma [53]. Human YTHDF1 will promote protein synthesis via interacting with post-transcriptional translation mechanisms. The relationship between YTHDF1 and eIF3 contribute to the link of YTHDF1 with the translation initiation complex and YTHDF1 can bind to specific m6A sites locateing at 3′UTRs, coding regions and 5′UTRs of mRNA to promote translation [54]. According to the Oncomine database, YTHDF1 was highly expression in tumors and it overall expression level was strong correlation with survival prognosis, tumor mutational burden (TMB), immune checkpoints (ICP), microsatellite instability (MSI) and neoantigens formation. Moreover, YTHDF1 also regulates tumor microenvironment (TME) and participates in immune regulation. We can trust that YTHDF1 might be a novel moderator in tumor immunotherapy [55]. The IGF2 mRNA-binding protein family (IGF2BPs) consists of two RNA-recognition motifs (RRM) and four KH domains guiding the cytoplasmic fate of various target mRNAs and controls essential cellular functions [56, 57]. Xie et al. found that circPTPRA could hinder the recognition of m6A-modified RNAs mediatd by IGF2BP1 through interacting with KH domains of IGF2BP1. Ectopic expression of circPTPRA will inhibit the facilitation of IGF2BP1 toward cell proliferation, migration and invasion of bladder cancer cells [58]. Recent publication indicated that IGF2BP2 induced the expression of ErbB2 by maintaining the m6A sites of YAP which further affected the cell cycle and cell apoptosis of colorectal cancer cell [59]. Based on the data of bio-informatics and experimental methods, IGF2BP3 is over-expressed in bladder cancer and was strongly correlated with patients’ survival prognosis. In addition, IGF2BP3 will activate the JAK/STAT pathway and then accelerate cell proliferation [60]. On conclusion, IGF2BP3 might be a potential therapeutic target of bladder cancer that can greatly change the prognosis of patients.

### Role of m6A modification in the EC

In 2019, Liu et al. found that FTO expression level was stronger in ESCC than that in compared normal tissues and the patients with higher expression of FTO frequently had worse prognosis. The western blotting indicated that FTO interacted with MMP13 and further study found FTO played oncogenic role needed stability expression of MMP13 [61]. Subsequent studies discovered that YTHDC2 expression level was lower in ESCC based on RNA-seq data consisting with TCGA database. Rs2416282 in the promoter of YTHDC2 might be a risk factor of ESCC. Knockdown YTHDC2 could enhance cell growth while elevated YTHDC2 expression could suppress cell proliferation, suggesting that YTHDC2 acts as a tumor suppressor in ESCC [62]. LINC00278 encoding Yin Yang 1 (YY1)-binding micropeptide (YY1BM) is a Y-linked lncRNA downregulated in ESCC. Cigarette smoking might decrease m6A modification sites of LINC00278 and synthesis of YY1BM. The translation efficiency of YY1BM could be enhanced by recruiting the translation machinery resulted from YTHDF1 binding with LINC00278. Mutual effection between YY1 and androgen receptor (AR) was suppressed by YY1BM and further induced the decreasing the eEF2K. Nevertheless, down-regulating YY1BM could confer ESCC more ability to nutrient deprivation through up-regulating eEF2K expression [63]. Nagaki et al. found ALKBH5 was correlated with poor prognosis in high expression level of ESCC patients demonstrating ALKBH5 might be a independent prediction factor of the survival ration. Cell cycle of ESCC cells will be delayed and accumulated to G0/G1 phase by knocking down ALKBH5. This resulted in suppression of the ESCC biological behaviour, such as proliferation and migration [64]. The IHC results showed that the higher expression of METTL3 was associated with the worse survival. Further studies found the METTL3 was positively correlated with the unfavourable survival indicating METTL3 might act as a symbol of disease-free survival (DFS) and overall survival (OS) [65]. Recently, interests focusing on function of non-coding RNA in EC became a hot topic. The underlying mechanism of miR-193a-3p has been uncovered that researchers find miR-193-3p suppresses the expression of ALKBH5. Furthermore, the miR-193-3p-ALKBH5 coupled loop could enhance cell invasiveness in vitro experiment [66]. In 2021, with the popularization of methylated RNA immunoprecipitation sequencing (MeRIP-Seq), researchers found METTL3 could affect glutaminase 2 (GLS2) via m6A modification. Chen et al. demonstrated that a direct strong binding interaction between GLS2 and METTL3 was detected using a RIP assay. They also discovered that knock-down of GLS2 expression level inhibited the invasiveness and migration capability of ESCC cells, indicating that GLS2 might be a carcinogenic risk in ESCC [67]. Wang et al. revealed a previously unknown mechanism that YTHDF/METTL3 together regulating m6A of APC mRNA would reduced APC expression. Promotion of β-catenin signaling pathway and accumulation of aerobic glycolysis have been confirmed through the aforementioned mechanism. The mechanism of METTL3/YTHDF coupled m6A regulation toward APC mRNA may provide therapeutic strategy for ESCC patients [68]. Hou et al. found that METTL3 could affect AKT signaling pathway and then alter the biological behavior of the EC [69]. Earlier studies found Colon cancer-associated transcript 2 gene (CCAT2) was high-expression in tumor and was associated with patient’s survival prognosis [70]. Wu et al. recently discovered CCAT2 could reduce miR-200b expression and further lead to IGF2BP2 high-expression. In turn, IGF2BP2 enhanced TK1 stability by identifying its m6A sites. On conclusion, this study proved that CCAT2 alleviate its suppression effects on IGF2BP2 by binding to miR-200b, resulting in upregulation of TK1 expression level and facilitation of ESCC [71]. Cellular lipid synthesis was inhibited by down-regulation of HNRNPA2B1 induced decreasing of fatty acid synthesis enzymes ACLY and ACC1. The result is that HNRNPA2B1 as a potential oncogenic factor could affect cell biological behaviour of ESCC [72]. Another researcher reported that HNRNPA2B1 was a prognosis predict factor of EC by influencing the miR-17-92 cluster [73]. AS for HNRNPC, HNRNPC can strengthen the stability of ZEB1 mRNA and ZEB1-induced high-expression of LBX2-AS1 enhanced migration and epithelial-mesenchymal transition capability of ESCC [74]. Currently, majority of investigations on m6A in the EC focused on the ESCC, but seldom on the EAC. Lately, we can see a lot of studies uncovering the mechanism about m6A and adenocarcinoma. And more exploration is needed in studying the relationship between EAC and m6A modification.

### The diagnosis potential of m6A for the EC

Early time, METTL3 up-regulated in tumor tissues was negatively related with patients’ DFS and OS. Liu et al. found 18F-FDG intake was increased along with high expression level of METTL3. The underlying mechanism of METTL3 increasing the intake of 18F-FDG might be controlled by GLUT1 and HK2. So PET/CT is a noninvasive monitoring to assess the condition of METTL3 [75]. Maybe we can use PET/CT and METTL3 coupled indicators as predictors of EC. Li et al. constructed a 4-miRNA survival predict model by combining the data of TCGA with GEO datasets [76]. With the in-depth of m6A research, m6A modification is expected to be an early detection biomarkers of the EC.

### The therapeutic potential of m6A for the EC

The traditional treatment modalities for the EC include operative treatment and radiotherapy as local therapies, while chemotherapy and targeted drug therapy as systemic treatments. In early stage of EC, a combination of endoscopic resection and adjuvant chemoradiotherapy is a therapeutic option with promising results. As for advanced ESCC, the aim of the treatment is to alleviate patient’s symptoms and prolong patient’s survival, therefore, chemotherapy and radiotherapy are usually adopted. The potential applications of targeted treatment based on m6A was concluded in previous studies. MO-I-500, a selected inhibitor of FTO, inhibited survival and colony formation of breast cancer cell lines SUM149-MA [77]. Metabolic reprogramming, driven by IGF2BP3, facilitates the development of acquired resistance to EGFR inhibitors in non-small cell lung cancer which suggests a novel perspective on the alteration of drug resistance [78]. Yankova et al. found treating tumours with STM2457, a METTL3 inhibitor, lead to reduced acute myeloid leukaemia (AML) growth and an increase in differentiation and apoptosis [79]. Other researchers also found that STM2457 can reverse small cell lung cancer chemoresistance by inducing mitophagy [80]. Anti-HIV Drug Elvitegravir enhanced the ubiquitination degradation of METTL3 by promoting the interaction between METTL3 and E3 ubiquitin ligase STUB1, which confirmed that the drug has a significant inhibitory effect on ESCC invasion and metastasis [81]. However, the development of inhibitors or activators of m6A-related proteins is still in the initial stage. Existing inhibitors or activators of m6A-related proteins generally have problems such as low activity, poor specificity, over-complex phenotype of intracellular effects. Therefore, m6A-related protein inhibitors or activators are mainly used in preclinical experimental studies and there is no researches applying inhibitors or activators of m6A-related proteins on ESCC patients. Some researches had reported that m6A overall expression level was up-regulated after Platinum-based chemotherapy. Platinum could induce SNHG3 expression but suppress microRNA-186-5p. The result is that m6A level is promoted by SNHG3 but inhibited by miR-186-5p through targeting METTL3. Zhang et al. put forward a hypothesis that regulating the overall m6A expression level might be an innovative strategy to improve the platinum treatment efficacy in EC patients [82]. Immune checkpoint therapy is a milestone in the field of cancer treatment. Through targeting PD-1 or PD-L1, the adaptive immune system can eliminate cancer cells and achieve a promising therapeutic efficacy. It is reported that FTO could promote melanoma tumorigenesis and decrease its response to immunotherapy in melanoma [83]. In recent years, immunotherapy achieved exactly clinical benefits in patients with ESCC. Guo et al. found that m6A influenced the micro-circumstances of tumor-infiltrating immune cells, suggesting that m6A could change the tumor immune microenvironment (TIME) [84]. Hence, m6A modification regulators is expected to elevate the efficacy of immunotherapy. Taken together, m6A methylation regulator not only adjust the PD-L1 expression level but also regulate the situation of immune cell infiltration. Therefore, a prognostic model including five m6A control elements (HNRNPC, RBM15, IGF2BP3, METTL16 and KIAA1429) was constructed to predict the prognosis of ESCC. From above researches, we hope that the m6A modification regulators may elevate the effect of immunotherapy.

## Discussion

Currently, the main treatments of the EC include surgery, radiotherapy, chemotherapy, target therapy and Immune checkpoint therapy. However, advanced esophageal carcinoma with high occurrences of metastasis and the overall survival is disappointment. It is crucial to clarify the specific mechanism of EC carcinogenesis. m6A modification is a new way of RNA manifestation. With the development of m6A profiling technologies, it is hoped that the pace of discovering the location of m6A modification will greatly accelerate. The m6A methylation modification has drawn more and more vision. Previous studies had explicit the mechanism of m6A modification in human cancer occurrence and development in kinds of cancer types such as liver cancer and non-small-cell lung cancer etc. In this research, the initiation and progression of the EC are highly associated with kinds of m6A regulators. METTL3 is not only up-regulated in the EC but also associated with a patient's DFS and OS. Nevertheless, ALKBH5 might act as a suppressor gene towards the EC, and patients with a lower expression level of ALKBH5 is usually accompanied by poor prognosis. Esophageal cancer tissues always with high-expression of HNRNPC and was correlated with poor prognosis of survival. From the above findings, we know that m6A methylation modification can regulate the rate of cell proliferation, affect distant of tumor cell metastasis, change ability of cancer cell invasion and alter the drug resistance of tumor cell.

The m6A regulators have the potential to be clinical treatment targets for different types of cancers. Rhein derived from the rhizome of Rheum palmatum was found to competitively bind to the specific FTO site [85]. R-2-hydroxyglutarate (R-2HG), produced by mutant isocitrate dehydrogenase 1/2 (IDH1/2) enzymes, exhibits a extensive anti-leukemic activity by inhibiting FTO activity and increasing m6A RNA modification in R-2HG-sensitive leukemia cells [86]. However, drug resistance or decreasing sensitivity to radiotherapy continues to be a dominant barrier to remedial treatment, leading to treatment failure and tumour progression. Su et al. found YTHDC1 regulates PTEN/PI3K/AKT signalling pathway and plays a critical role in cisplatin resistance of bladder cancer [87]. In addition, METTL3 improves the progression of pancreatic ductal adenocarcinoma and resistance to gemcitabine by modifying m6A of DDX23 mRNA [88]. Based on the literature above, we can see that m6A is very promising for enhancing drug resistance. NRP1, a transmembrane glycoprotein, contributes to stemness and radioresistance of breast cancer through WTAP-mediated m6A methylation of Bcl-2 mRNA [89]. Wu et al. discovered that METTL3 mediating the m6A methylation of circCUX1, a specific circRNA, lead radioresistance of hypopharyngeal squamous cell carcinoma through caspase1 pathway [90]. These articles provide new clues about the therapeutic direction of m6a in radiotherapy (Table 1).

**Table 1:** The role of different m6A regulators in esophageal carcinoma

m6A regulators	Genes	Location	Role	Mechanism	Function
Writer					
METTL3	–	–	Oncogene	Activating the Wnt3/β-catenin and AKT signaling pathways	Promoting ESCC cell proliferation
	APC	mRNA	Tumor suppressor	Reducing APC expression	Promoting ESCC cell proliferation and tumour development
	GLS2	mRNA	Oncogene	GLS2 as a downstream Target of METTL3	Promoting ESCC cell migration and invasion
	SNHG3 and miR-186-5p	mRNA and micro-RNA	–	SNHG3/miR-186-5p induced by platinum, was involved in regulating m6A level by targeting METTL3	Regulating m6A level might be a novel way to enhance the platinum efficacy
	IFIT2	mRNA	Tumor suppressor	IFIT2 mRNA and protein expression were both downregulated by METTL3 overexpression	IFIT2 overexpression Inhibiting ESCC cell proliferation and invasion
	TNFR1	mRNA	Oncogene	TNFR1 regulates the activation of MAPK and NF-κB signaling pathways	The METTL3-m6A-TNFR1-ATXN2 axis plays oncogenic roles in ESCC through MAPK and NF-κB signaling pathways
	miR-320b	Micro-RNA	Oncogene	METTL3 could interact with DGCR8 protein and positively modulate pri-miR-320b maturation process in an N6-methyladenosine (m6A)-dependent manner	miR-320b promotes the proliferation, migration, invasion, and epithelial-mesenchymal transition progression of ESCC cells
	miR-20a-5p	Micro-RNA	Oncogene	METTL3 promote m6A modification and the binding of DGCR8 to miR-20a-5p to further elevate the miR-20a-5p expression and inhibit NFIC transcription	miR-20a-5p upregulation facilitates ESCA cell invasiveness and migration by targeting Nuclear Factor I-C(NFIC) transcription
	NOTCH1	mRNA	Oncogene	NOTCH1 signaling pathway is an important downstream target of METTL3 and is essential for its function in promoting ESCC progression	METTL3-catalyzes m6A modification promotes NOTCH1 expression and the activation of the Notch signaling pathway
	EGR1	mRNA	Oncogene	METTL3 increases m6A in EGR1 mRNA and enhances its stability in a YTHDF3-dependent manner	METTL3 promotes cancer metastasis by activating EGR1/Snail signaling in an m6A-dependent manner
	COL12A1	mRNA	Oncogene	COL12A1 serves as a potential target gene of METTL3 and acts as an oncogene in the progression of ESCC	METTL3 enhanced proliferation and metastasis of ESCC through COL12A1/MAPK signaling pathway
METTL14	miR-99a-5p	micro-RNA	Tumor suppressor	METTL14 upregulates miR-99a-5p by modulating m6A-mediated, DiGeorge critical region 8-dependent pri-mir-99a processing	METTL14/miR-99a-5p/TRIB2 axis shows that it is positively associated with cancer stem-like cells characteristics and radioresistance of ESCC
Eraser					
FTO	MMP13	mRNA	Oncogene	Stabilizing MMP13 mRNA	Enhancing ESCC cell viability and migration
	HSD17B11	mRNA	Oncogene	FTO promote the formation of lipid droplets in EC cells by enhancing HSD17B11 expression	High expression level of HSD17B11 promoting the aggregation of lipid droplets
	ERBB2	mRNA	Oncogene	ERBB2 is the target gene of FTO in ESCC cells	YTHDF1 stabilizes ERBB2 mRNA via decoding the m6A modification and ERBB2 involves in the tumorigenesis of ESCC progression
	LINC00022	IncRNA	Oncogene	FTO in ESCC decreased m6A methylation of LINC00022 transcript, leading to the inhibition of LINC00022 decay via the m6A reader YTHDF2	LINC00022 directly binds to p21 protein and promotes its ubiquitination-mediated degradation, thereby facilitating cell-cycle progression and proliferation
ALKBH5	CDKN1A	mRNA	Tumor suppressor	Stabilizing CDKN1A mRNA	Promoting ESCC cell proliferation
	miR-193-3p	micro-RNA	Oncogene	inhibiting miR-193a-3p expression	Promoting the proliferation, migration and invasion ability of ESCC cells
	miR-194-2	micro-RNA	Oncogene	ALKBH5 regulates RAl1 by reducing miR-194-2-mediated RAl1 suppression	ALKBH5 suppresses esophageal cancer malignancy by inhibiting m6A/DGCR8-dependent miRNA biogenesis and unleashing RAl1 expression
Reader					
WTAP	CPSF4	mRNA	Tumor suppressor	WTAP mediated m6A of CPSF4 mRNA in an YTHDF2-dependent manner	Decreasing CPSF4 expression in an m6A-dependent manner facilitates ESCC tumour growth and metastasis
IGF2BP2	LNC-CCAT2	IncRNA	Oncogene	Inhibiting miR-200b to upregulate the IGF2BP2/TK1 Axis	Ensuing promotion of the development of ESCC
IGF2BP2 and IGF2BP3	LINC01305	IncRNA	Oncogene	linc01305 promotes HTR3A mRNA stability through interacting with IGF2BP2 and IGF2BP3	HTR3A promoting migration and proliferation of ESCC
YTHDF1-3	APC	mRNA	Tumor suppressor	Mediating APC mRNA degradation	Promoting β-catenin-mediated downstream gene expression, aerobic glycolysis and ESCC cell proliferation
YTHDF1	LINC00278	IncRNA	Tumor suppressor	Promoting the translation efficiency of YY1BM	Promoting apoptosis of ESCC cell
	HLA complex P5 (HCP5) and HK2	Endogenous retroviral gene	Oncogene	Promoting the Warburg effect (aerobic glycolysis) of ESCC cells	The turbulence of HCP5/YTHDF1/HK2 axis may be responsible for ESCC carcinogenicity
YTHDC2	rs2416282	SNP	Tumor suppressor	Increasing YTHDC2 expression by allele-specific binding to transcription factors	rs2416282 reducing ESCC risk in Chinese population by altering the expression of YTHDC2
HNRNPA2B1	ACLY and ACC1	Enzymes	–	Up-regulating the fatty acid synthesis enzymes ACLY and ACC1	Promoting ESCC progression via up-regulation of fatty acid synthesis enzymes ACLY and ACC1 and cellular lipid accumulation
	miR-17-92 cluster	Micro-RNA	ONCOGENE	Correlating with the cell cycle and RNA transport signaling pathways	HNRNPA2B1 affects tumor-promoting signaling pathways by regulating the expression of the miR-17-92 cluster
HNRNPL	CASC8	lncRNAs	Oncogene	CASC8 interacte with heterogeneous nuclear ribonucleoprotein L (hnRNPL) and inhibited its polyubiquitination and proteasomal degradation, thus stabilizing hnRNPL protein levels and activating the Bcl2/caspase3 pathway	CASC8 decreases the cisplatin sensitivity of ESCC cells and promoted ESCC tumor growth

Previous studies have validated that the m6A score could be a credible biomarker for predicting the efficacy of anti-PD-1/L1 immunotherapy in different types of tumours such as melanoma, breast cancer and colorectal cancer. In melanoma, lower m6Ascores always correlated with more sensitive anti-PD-1 and anti-CTLA4 treatment responses [91]. Some researchers have confirmed that the specific suppression of METTL3 myeloids could attenuate the inhibitory treatment of PD-1 by affecting the reprogramming of macrophages [92]. The potential mechanism of m6A in the immunotherapy of esophageal cancer remains unclear. Checkmate577, an adjuvant nivolumab therapy research, demonstrates for the first time that adjuvant immune checkpoint inhibitors therapy can lead to clinically significant improvements in disease-free survival in patients with resectable esophageal cancer and gastroesophageal junction cancer [93].This study serves as a precursor to the implementation of immunotherapy in the treatment of esophageal cancer. The prognostic survival of patients is closely associated with the tumor immune microenvironment. Nie et al. used single-cell mapping and immune infiltration risk model to estimate the m6A epigenetic-based riskscore [94]. They found ESCC patients with higher risk scores have lower expression levels of major immune cells such as in natural killer T cell (NKT), CD4 + naive T cells, M1 macrophages, ADC, and macrophages. METTL3 could regulate esophageal cancer proliferation, invasion and immunity via the downstream target IFIT2. TIMER database revealed a significant correlation between the expression of METTL3 and the degree of infiltration by B cells and macrophages [95]. The mechanism of mettl3 in the immunotherapy of esophageal cancer needs to be further explored. Moreover, Paclitaxel sensitivity is lower in the mutant CSMD1 group and CSMD1 mutation is associated with tumor invasion of immune cells with more follicular helper T cells and fewer resting state dendritic cells. This phenomenon suggests that CSMD1 mutation may serve as an immune-related biomarker for predicting the ESCC patient’s prognosis and treatment response to paclitaxel [96].m6A modification study progressing is still at the initial stage and there are still many challenges. The drug targeting specific m6A proteins is needed to design via high throughput drug screening and structural studies. We hope that m6A modification inhibitors will become a promising therapeutic methods for patients suffering different cancers. Scientists are devoted to inventing m6A modification detection kit for specific cancer to diagnosis in its early stage and developing particular reagent targeting m6A proteins to obtain good therapeutic effect.
